# Elevation in and persistence of multiple urinary biomarkers indicative of oxidative DNA stress and inflammation: Toxicological implications of maleic acid consumption using a rat model

**DOI:** 10.1371/journal.pone.0183675

**Published:** 2017-10-26

**Authors:** Charlene Wu, Hsin-Chang Chen, Shu-Ting Chen, Su-Yin Chiang, Kuen-Yuh Wu

**Affiliations:** 1 Institute of Occupational Medicine and Industrial Hygiene, College of Public Health, National Taiwan University, Taipei, Taiwan; 2 National Environmental Health Research Center, National Health Research Institutes, Zhunan, Miaoli, Taiwan; 3 School of Chinese Medicine, China Medical University, Taichung, Taiwan; Kermanshah University of Medical Sciences, ISLAMIC REPUBLIC OF IRAN

## Abstract

Maleic acid (MA), an intermediate reagent used in many industrial products, instigated public health concerns in Taiwan when it was used to adulterate an array of starch-based delicacies to improve texture and storage time. Established studies reported that exposure to high concentrations of MA induce renal injury; little is known whether oxidative stress is induced at a relative low dose. This study aims to investigate the effect of oral single dose exposure of MA on the status of oxidative stress and inflammation. Single dose of MA at 0, 6 and 60 mg/kg (control, low- and high-dose groups, respectively) were orally administered to adult male and female rats. Urine samples were collected and analyzed to measure 8-hydroxy-2’-deoxyguanosine (8-OHdG), 8-iso-prostaglandin F_2α_ (8-IsoPGF_2α_), 8-nitroguanine (8-NO_2_Gua) and N-acetyl-S-(tetrahydro-5-hydroxy-2-pentyl-3-furanyl)-L-cysteine (HNE-MA) using LC-MS/MS. Results revealed that oral consumption of MA induced oxidative DNA damage and lipid peroxidation, as demonstrated by the statistically significant increases in urinary levels of 8-NO_2_Gua, 8-OHdG, and 8-isoPGF_2α_, in high-dosed male rats within 12 h of oral gavage (*p* < 0.05). Additionally, increases in concentration of these biomarkers persist for days after consumption; male rats appear to be more sensitive to oxidative burden compared to their counterparts. The aforementioned findings could help elucidate the mechanisms through which nephrotoxicity occur.

## Introduction

Intentional adulteration of Maleic anhydride (MAH), an organic and multifunctional chemical intermediate used in many fields of applied chemistry, in many foods raised health-related anxiety in both Taiwan and abroad. Maleic acid (MA), the hydrolyzed form of MAH, typically functioned as a pH adjuster, a fragrance ingredient at low concentrations, as well as an adhesive in endodontics [1]. Thus, the general population is likely to be exposed to MA upon contact from personal care products and through inhalation of dust and aerosols from automobile emissions [2, 3]. The California Environmental Protection Agency estimated the statewide emission rate of MAH from industrial facilities at 3340 kg year^-1^. Upon dermal contact and via inhalation, MA can irritate the mucous membranes of the eye and the upper respiratory tract, respectively, at as low as 0.25 ppm [4].

Toxicity studies on MA revealed a broad range of symptoms in various animal hosts. Exposure to high doses of MA could result in phenotypic manifestations such as alopecia and significant decreases in both absolute and relative organ weights [5]. To achieve detection of low concentrations of MA, advances in analytical methods with LC-MS/MS and Near-Infrared (NIR) spectroscopy and chemometrics were reached to attain high sensitivity and specificity [6, 7]. Improvements in these methods furthered our understanding of the pharmacokinetic behavior and can also assist in investigating the toxicological implications of MA from consumption [8–10]. However, most of the toxic effects occur at the cellular and tissue levels. Few studies have explored the relationship between MA exposure and oxidative stress, which occurs when the formation of ROS overpowers cellular antioxidant defenses and exert cytotoxicity by damaging cellular constituents. Existing literature note that once consumed, absorbed and metabolized, MA can penetrate kidney cells to incur renal injury, which is associated with elevated oxidative stress status [11]. When MA is injected into rodents and once intracellular accumulation occurs, maleate becomes the preferred substrate for succinyl-Coenzyme A (CoA):3-oxoacid CoA transferase (SCOTase). This newly formed maleyl-CoA, when transforms into a stable thioether, results in CoA and adenosine triphosphate (ATP) depletion [12–15]. MA was also observed to conjugate the sulfhydryl group of glutathione (GSH) thus depleting GSH; previous reports speculated such depletion inhibits glutathione peroxidase activity, increases lipid peroxidation in renal tissue and may also induce potential does-dependent oxidant proximal tubule (PT) injury [16–18]. Available research indicated that rats treated with maleate toxicity increases the production of heme oxygenase 1 (HO-1), which is generated in response to oxidant stress [16, 19]. Filtration failure and proximal tubular necrosis could result depending on the severity of these processes. However, others noted that maleate-induced ATP depletion, and not oxidative stress, is responsible for proximal tubule injuries [20]. This concept is further supported by Zager, as no evidence of maleate-induced stress responses were noted in trial exposures [21]. Therefore, current literature has yet to agree on whether MA induces oxidative stress, lipid peroxidation and inflammatory response on a cellular level.

Oxidative stress is also reported to correlate with the onset or progression of an array of ailments, including cancer, interstitial lung disease and acute renal ischemia [22, 23]. 8-OHdG, induced by ROS, is a biomarker indicative of oxidative DNA impairment and has utility in predicting renal damage [24]. 8-NO_2_Gua, a potential biomarker for nitrative DNA damage, is also shown to be indicative of inflammatory response [25]. Furthermore, 8-OHdG and 8-NO_2_Gua have also been recognized as indicators of cellular mutagenicity [22, 26]. The F2-isoprostanes is another sensitive and reliable indicator of oxidant burden. Among them, many studies indicated 8-isoPGF_2α_ as reliable indicator of lipid peroxidation because of its stability. Additionally, elevated levels of 8-isoPGF_2α_ have been noted in association with exposure to toxic compounds and may thus indicate disease progression in the lungs and kidneys [27–33]. Moreover, HNE-MA, a biomarker detected in rats after an acute oxidative stress insult, can indicate levels of lipid peroxidation and be used to evaluate cytotoxicity in biological systems [34].

In light of the inconsistent reports regarding whether MA exposure induces oxidative stress, inflammation, and lipid peroxidation, coupled with the extreme difficulty in determining ROS/RNS in tissues or body fluids due to their high reactivity and extremely short half-lives, this study aims to determine, via LC-MS/MS, whether single-dose exposure of MA affects the urinary levels of the four aforementioned biomarkers. To our knowledge, no study has yet analyzed whether consumption of MA induce oxidative burden. This study also investigated the histological alterations that may precipitate from such exposure. The results of our study would not only clarify the relationship between MA exposure and oxidant stress status, but also broaden our understanding of the mechanisms through which organ damage occur at a cellular level.

## Materials and methods

### Chemicals and reagents

HNE-MA (1mg in 100 μl ethanol), HNE-MA-d_3_ (100 μg in 100 μl ethanol), 8-isoPGF_2α_, and 8-isoPGF_2α_-d_4_ were purchased from Cayman Chemicals (Ann Arbor, MI, USA). Maleic anhydride, 8-OHdG, and 7.5M of ammonium acetate solution (NH_4_Ac_(aq)_), 0.1 N sodium hydroxide (NaOH) standard solutions, and were purchased from Sigma–Aldrich (St. Louis, MO, USA). The internal standard, ^15^N_5_-8-OHdG (99% purity), was acquired from Cambridge Isotope Laboratories (Andover, MA, USA). Unlabeled 8-NO_2_Gua and its internal standard, 8-NO_2_Gua-4, 8-^13^C_2_-7-^15^N, were obtained from Santa Cruz Biotech (Santa Cruz, CA, USA). HPLC-grade methanol (MeOH) was procured from MACRON Chemicals (Center Valley, PA). For all subsequent steps, Milli-Q water was produced by a Millipore Elix 10 RO system and a Millipore Synergy UV system (Millipore SAS, Molsheim, France).

### Stock solutions and working solutions

All stock solutions were preserved at -20°C. The stock solutions of 8-OHdG and 8-isoPGF_2α_ (both at 10 μg/ml) were prepared by dissolving the solid standards in methanol and 0.1 M NaOH aqueous solution, respectively, to reach a concentration of 10 μg/ml. To construct a calibration curve in the aqueous solution, six working standard solutions containing the four analytes were prepared by serial dilutions in deionized water to final concentrations covering a range of 0.1 to 50 ng/ml. Each working solution contained the four internal standards at a fixed amount of 10 ng/ml, and each calibration point was determined from the average of three replicate measurements.

### Animal treatment and sample collection

Equal numbers of male and female SD rats (*n* = 15 per sex; body weight = 215 g to 250 g for males; BW = 185 g to 210 g for females) were purchased from BioLasco Taiwan Co., Ltd. (Yilan, Taiwan). Animals were allowed to acclimate for a week prior to the initiation of treatment, during which time they were weighted and checked daily for dietary and excretory conditions. Animals were housed in a temperature- (22 ± 1°C) and humidity- (45 ± 5%) controlled room with a 12-hour light/dark cycle. All rats’ feed and water were provided separately and *ad libitum*; with purified water (18.2 MΩ·cm) supplied as source of drinking water. The Institutional Animal Care and Use Committee (IACUC) of China Medical University (No. 102-258-N) deemed the treatment of these animals ethical and approved this investigation.

Animals were allocated to dosage groups based on the stratified weight to achieve identical group weight averages. On the day of oral gavage (prior to administration), the average BW of male rats ranged from 231.6 g to 234.4 g; average BW of female rats ranged from 194.6 g to 205.6 g. Rats were treated via oral gavage with single dose at 0 mg/kg (control group), 6 mg/kg (low-dose group), and 60 mg/kg (high-dose group) of MA with distilled deionized water as the vehicle. Before treatment, urine samples were collected for analyzing background levels of MA. After dosing, rats were immediately placed into metabolic cages; daytime urine samples were collected, with 50-mL centrifuge tubes containing 100 μL of 5% sodium azide, at 0.5, 1, 2, 3, 4, 5, 6, 7 d after initial oral gavage. Post collection, urine volumes were recorded and samples were centrifuged at 3420 × *g* using a Universal 320R centrifuge (Andreas Hettich GmbH & Co. KG, Germany) for 5 min; all samples were stored at -80°C until analysis. All animals were sacrificed by decapitation at the end of the trial.

### Measurements of urinary biomarkers

The collected urine samples were prepared and analyzed according to our previously published protocol with slight modification [35]. In brief, samples were thawed at room temperature, vigorously vortexed and centrifuged at 10,000 rpm for 10 min. Subsequently, 50 microliters of urine supernatant was pipetted into an Eppendorf tube, diluted 20-fold with deionized water containing 1 mM of ammonium acetate, spiked with 10 μl of isotopically labeled standards (100 ng/ml), and vortexed again for solid-phase extraction (SPE), which was carried out by using an Oasis HLB cartridge (1 cc, 30 mg; Waters, Milford, MA, USA). The cartridge was preconditioned with 2 ml of methanol and 2 ml of water. After sequential loading, the cartridge was washed with 2 ml of water. Subsequently, the analytes were eluted with 1 ml 100% (v/v) MeOH. The solutions were evaporated to dryness with a rotary evaporator (Savant SPD131DDA SpeedVac Concentrator, Thermo scientific); subsequent residue was re-dissolved in 200 μl of 5% (v/v) MeOH containing 1mM of NH_4_Ac_(aq)_. An aliquot of 25 μl was injected into the LC-MS/MS system for quantitation.

Quantitation and qualification were performed with the HPLC system comprised a quaternary pump (Accela micropump, Thermo Fisher Scientific), an autosampler (Accela, Thermo Fisher Scientific), and a 3 μm, 100x2 mm Gemini-NX-C18 analytical column (Phenomenex, Torrance, CA). HPLC was executed with mobile phase A, comprised of 95% MeOH and 1 mM NH_4_Ac_(aq)_ (Methanol:1mM ammonium acetate = 95:5, v/v) and mobile phase B, which consist of 5% MeOH and 1 mM NH_4_Ac_(aq)_ (Methanol:1mM ammonium acetate = 5:95; v/v). With the flow rate set at 150 μl/min, the linear gradient was modified to the following: held constant at 1%A for 2 min; running from 1 to 40% A for 0.5 min, 40 to 75% A over the next 3.5 min, and 97% A for 1 min; increasing to 99% A for next 1.5 min; held constant for 2 min; returned to 1% A for 0.5 min; and held constant for 2 min to reach equilibrium.

**Fig 1:**
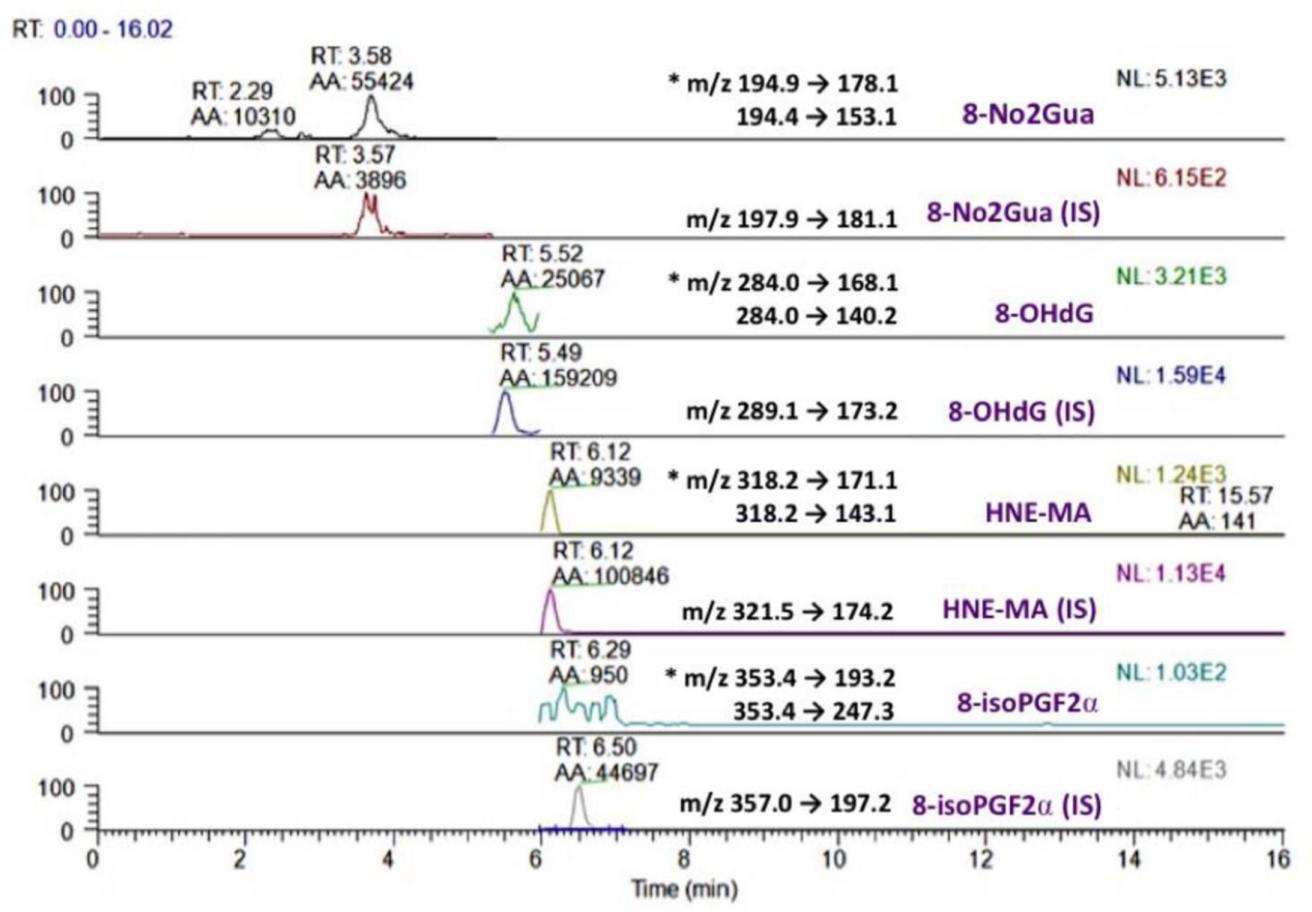
Chromatogram of rat urine samples using offline SPE-LC-MS/MS; the quantitation channels are marked with *.

Analytes were detected with a triple-quadrupole tandem mass spectrometer (TSQ Quantum Access, Thermo Fisher Scientific, USA) equipped with a heated ESI source. To accomplish separation analysis under different detection modes, we used Xcalibur software (version 2.0.7, Thermo Fisher Scientific, USA), to divide the analytical process into several segments. With the exception of unlabeled and isotopically labeled 8-OHdG, all other analytes (8-NO_2_Gua, 8-isoPGF_2α_, HNE-MA) and their internal standards were detected under the negative ion mode. Several parameters were optimized to the following: For the analysis of 8-NO_2_Gua, 8-isoPGF_2α_ and HNE-MA, spray voltage was set at 2500V, the vaporizer temperature was maintained at 100°C, and the capillary temperature was set at 250°C. The sheath and auxiliary gas pressures were set at 30 and 5 psi, respectively. The argon gas pressure was set at 1.5 mTorr. For the quantitation of 8-OHdG, the optimized spray voltage was set at 3000 V, and the vaporizer and capillary temperatures were maintained at 100 and 210°C, respectively. In addition, the sheath and auxiliary gas pressures were set at 45 and 15 psi, respectively. The collision gas pressure set at 1.5 mTorr. Fig 1 displays the successful simultaneous quantitation of the abovementioned analytes with their respective internal standards.

### Measurements of urinary creatinine

Urinary creatinine was used to normalize the measured levels of the four biomarkers, and the creatinine concentration was measured as the creatinine-picrate complex with a U-2000UV/VIS spectrophotometer (Hitachi, Tokyo, Japan) at a wavelength of 520 nm. The urinary levels of each analyte were expressed as μg/g creatinine.

### Statistical analyses

One-way ANOVA for multiple comparisons and Student's t-test for unpaired data were calculated using SAS 9.2 to determine statistical significance. Results were considered statistically significant at p <0.05 and <0.01.

## Results

### Effect of maleic acid on body weight

On the day of exposure, the average body weights of SD male rats in the control, low- and high-dosed groups were 211.0, 213.0 and 214.6 g, respectively (shown in Table 1). After the 7-day single-dose study, the average body weights of control group become 263.2 g and those of low- and high-dosed groups were 235.8 and 239.1 g, respectively. Slowing down of increases in average body weight of the low- and high-dosed group was statistically significant (*p* = 0.023 and 0.01, respectively). Furthermore, average body weights of the female MA-treated rats did not demonstrate significant fluctuations compared to those of male group (displayed in S1 and S2 Figs).

**Table 1:** Body weight change in maleic acid-treated SD rats.

Group (mg/kg)	Day 0 (BW in g)	Day 1 (BW in g)	Day 2 (BW in g)	Day 3 (BW in g)	Day 4 (BW in g)	Day 5 (BW in g)	Day 6 (BW in g)	Day 7 (BW in g)
**Male**								
**0**	233.4^a^ ± 7.348^b^	211.0 ± 8.354	234.4 ± 8.203	218.0 ± 8.093	240.2 ±10.40	255.0 ±12.30	254.8 ± 11.28	263.2 ± 9.549
**6**	231.6 ± 11.59	213.0 ± 12.02	235.0 ± 16.66	221.4 ± 13.46	244.6 ± 11.33	255.2 ± 16.69	258.4 ± 19.82	*235.8 ±19.63
**60**	234.4 ± 11.57	214.6 ± 11.33	241.6 ± 10.17	226.6 ± 11.15	243.4 ± 11.67	259.0 ± 13.37	264.6 ± 13.18	*239.1 ± 13.32
**Female**								
**0**	195.0^a^ ± 3.768^b^	191.8 ± 10.32	191.2 ± 7.190	196.6 ± 7.956	198.6 ± 9.044	204.0 ± 8.573	207.2 ± 9.091	206.2 ± 7.949
**6**	205.6 ± 17.40	196.4 ± 13.05	196.6 ± 14.47	201.0 ± 14.97	207.6 ± 14.94	213.8 ± 14.72	217.4 ± 19.07	215.0 ± 19.27
**60**	194.6 ± 5.549	176.2 ± 3.153	190.8 ± 4.147	191.8 ± 4.266	196.8 ± 9.471	197.4 ± 12.72	198.4 ± 13.48	199.4 ± 2.792

BW: body weight

^a^Mean of observed values (*n* = 5)

^b^Standard deviation of observed values (*n* = 5)

*statistically significant at *p* <0.05

### Effect of maleic acid on the urinary biomarkers

#### Male rats

**Fig 2:**
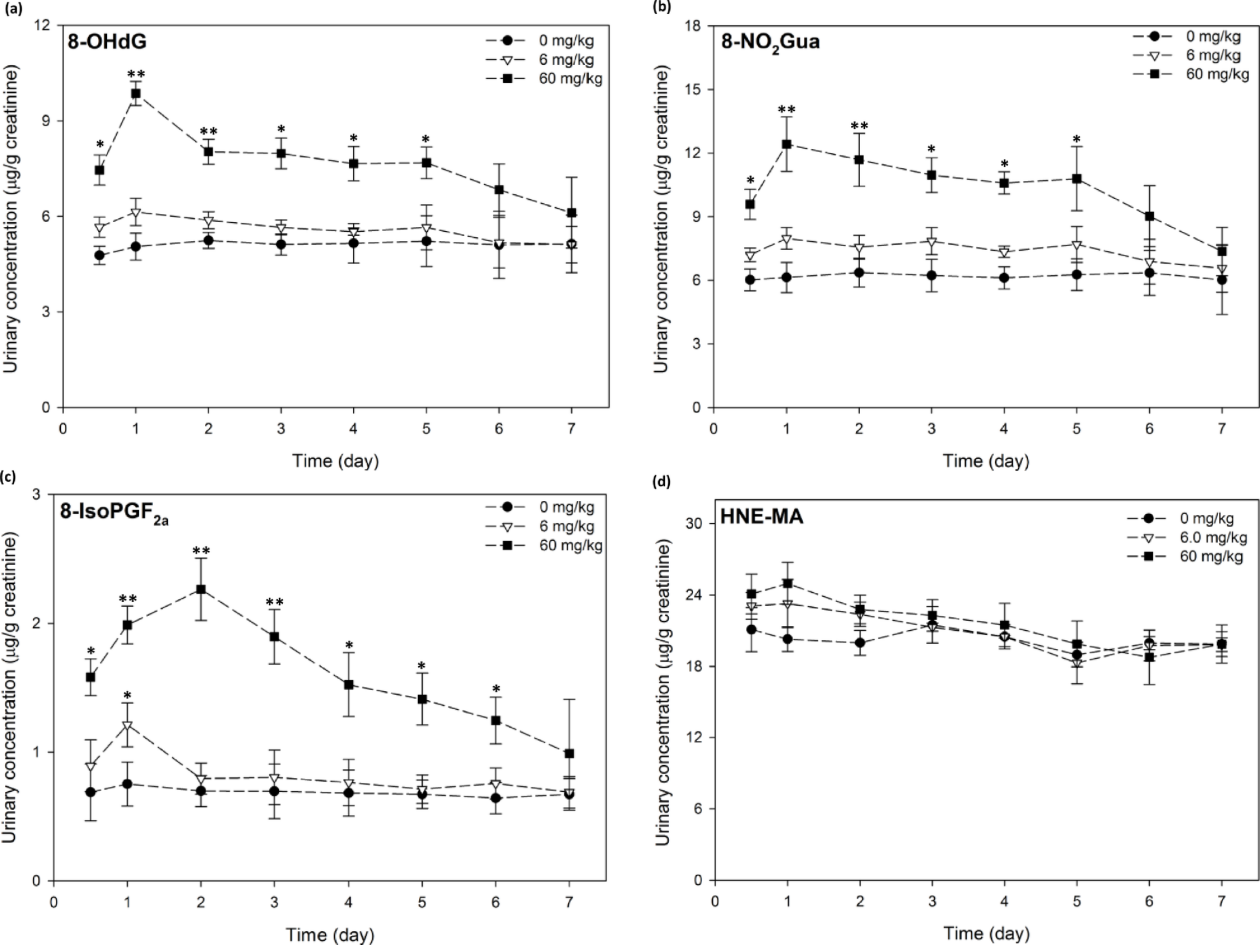
Excretion profiles of four oxidative biomarkers after single-dose exposure to maleic acid in male rats. (a) 8-OHdG levels remain elevated and persist until day 5; (b) 8-NO_2_Gua levels display similar trend to that of 8-OHdG; (c) 8-isoPFG_2α_ levels demonstrate elevation and persistence until day 6; (d) HNE-MA levels remain less affected after single-dose exposure to maleic acid. *, ** indicate statistically significant at *p*<0.05 and 0.01, respectively. Dose groups receiving 0 mg/kg (●), 6 mg/kg (○),and 60 mg/kg (▼) are denoted accordingly.

This study presented 7 day-excretion profile of the four urinary biomarkers for the control and treated male rats (Fig 2A–2D). 24 h after MA exposure, our results demonstrated that marked differences in urinary level of 8-OHdG was observed in the high-dosed SD rats when compared to that of the untreated rats (Fig 2A). Such significant increase in response persisted from 0.5 to 5 d post dosing. The highest observed urinary concentration of 8-OHdG was 9.87 ± 0.38 μg/g creatinine, which occurred at 24 h post exposure in the high-dose group, with the increase in urinary 8-OHdG in the high-dose group attaining statistical significance at the 0.01 level (*p* = 0.003).

The observed elevation and persistence in the urinary concentration of 8-NO_2_Gua was similar to that of 8-OHdG; in high-dosed male rats, the urinary concentration of 8-NO_2_Gua peaked at 24 h of dosing (12.42 ± 1.29 μg/g creatinine), attained statistical significance at the 0.01 alpha level, and gradually decreased to levels comparable to that detected in the low-dose and control groups (Fig 2B).

Urinary 8-isoPGF_2α_ concentrations for the control and low-dosed groups peaked at 0.75 ± 0.17 and 1.21 ± 0.17 μg/g on day 1 (Fig 2C); such increase did not attain statistical significance (p = 0.073). For the high-dose group, highest mean concentrations occurred on day 2 at 2.26 ± 0.24 μg/g with a *p*-value less than 0.01 (p = 0.006). Notably, levels of 8-isoPGF_2α_ remained elevated until day 6. Furthermore MA exposure did not appear to induce meaningful fluctuations in urinary HNE-MA levels in either treatment groups (Fig 2D). In the low- and high- dosed groups, peak concentrations occurred on day 1 at 23.29 ± 3.03 and 24.98 ± 3.77 μg/g. In general, concentrations of the four biomarkers in the control rats were lower than those in the treatment groups and maintained stable over the course of the study.

#### Female rats

**Fig 3:**
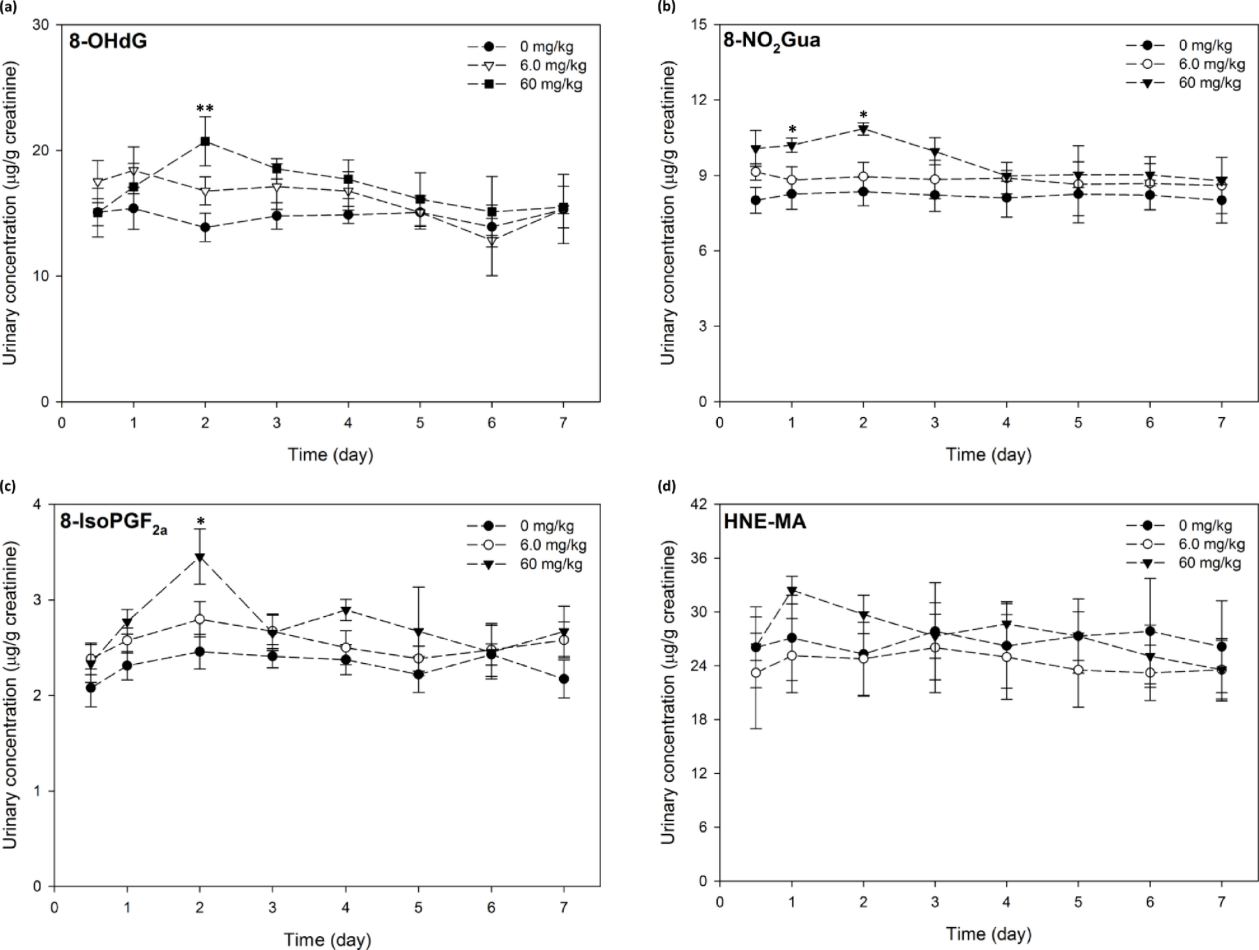
Excretion profiles of four oxidative biomarkers after single-dose exposure to maleic acid in female rats. (a) 8-OHdG levels remain slightly elevated over the course of the study; (b) 8-NO_2_Gua levels display similar trend to that of 8-OHdG; (c) 8-isoPFG_2α_ levels demonstrate statistical significant elevation on day 2; (d) HNE-MA levels remain less affected after single-dose exposure to maleic acid. *, ** indicate statistically significant at *p*<0.05 and 0.01, respectively. Dose groups receiving 0 mg/kg (●), 6 mg/kg (○),and 60 mg/kg (▼) are denoted accordingly.

Concentrations of 8-OHdG, 8-NO_2_Gua and 8-isoPGF_2α_ and HNE-MA all peaked within 48 h of exposure to MA (Fig 3A–3D) with minor fluctuations detected for the duration of the study. In general, our data demonstrated dose-dependent responses in urinary levels of 8-OHdG, 8-NO_2_Gua and 8-isoPGF_2α_; which was also observed in male rats (Fig 3A to 3C). Moreover, variations in the levels of the four biomarkers in the low-dose group were not statistically meaningful. For 8-OHdG, maximum mean concentrations of 8-OHdG were detected on day 1 (low-dose) and 2 (high-dose) after dosing. Our results demonstrated that the increase in 8-OHdG levels in the high-dose group reached statistical significance at day 2 (*p* < 0.01).

In high-dosed group, urinary 8-NO_2_Gua concentrations peaked on day 2 and decreased gradually to levels similar to that of low-dose and untreated rats by day 7. Statistically significant differences (*p* <0.05) in urine concentrations of 8-NO_2_Gua as compared to the controls were sporadic, only on day 1 and day 2 (Fig 3B). Much like that exhibited in the male rats, the shifts in the urine concentrations after MA exposure did not reach significance level denoted at α = 0.05. For 8-isoPGF_2α_, the increases in urinary levels in both dose groups occurred 2 days after dosing. In particular, the observed maximum mean concentration of 8-isoPGF_2α_ from the high-dose group was statistically significant at the 0.05 alpha level. We observed that for rats exposed to MA, concentrations of these three aforementioned biomarkers decreased gradually over time; only that of 8-OHdG remained distinctly and consistently higher than those measured in the control group. None of the fluctuations in urinary concentrations of HNE-MA reached statistical significance at both 95 and 99% confidence intervals.

## Discussion

The main objective of this study was to use a sensitive and non-invasive analytical method to investigate the effect of MA consumption on the formation of biomarkers indicative of oxidative stress (8-OHdG), inflammation (8-No_2_Gua) and lipid peroxidation (8-isoPGF_2α_ and HNE-MA). These aforementioned burdens can eventually result in cellular or tissue damage, manifested as cell necrosis or apoptosis [36–38]. Recently, Tapia and associates demonstrated that single intraperitoneal injection (i.p.) of MA, at 400 mg/kg, induced oxidative stress in kidney tissues; however, no other doses nor routes of administration was investigated [39]. To our knowledge, this present study is the first to examine whether oxidative burden occurs after single oral low-level exposure to MA at relatively low doses. Our study demonstrated that, with the exception of HNE-MA, statistically significant elevations in urinary levels of 8-OHdG, 8-NO_2_Gua and 8-isoPGF_2α_, which suggest that detoxification of MA induced oxidative burden and inflammatory responses through the production of ROS and RNS.

With the kidneys as the known major target organs of MA toxicity, several *in vitro* and *in vivo* studies have postulated the mechanisms through which renal damages occur. Some demonstrated that the preferential transportation of MA via organic anion transporters (OAT), and the formation of maleyl-CoA both induce dose-dependent ATP depletion, thus result in damages to the proximal tubule and renal ischemia [23, 40]. An alternate concept state that maleate toxicity stems partially from the build-up of oxidative stress, which is due to MA’s ability to deplete GSH [20, 41, 42]; other studies note that MA exposure leads to the formation of heme oxygenase-1, an enzyme formed as a response to oxidative stress [16, 19]. Given these discrepancy, our findings lend support to the latter toxicological mechanism of action since rat urine analysis confirmed statistically significant elevated urinary levels of 8-OHdG, the oxidative DNA lesions formed when ROS react with 2’-deoxyguanosine, as well as 8-NO_2_Gua, a nitrative DNA adduct caused by reactive nitrogen species and a reliable inflammatory biomarker [43–45].

Previously, our group published the pharmacokinetic study of MA upon oral administration [8]. In that study, MA is rapidly absorbed and metabolized upon consumption, with the majority of administered dose excreted through urine. Our current results suggest that even after MA has been absorbed and excreted, the elevated levels of the abovementioned biomarkers persisted for days after dosing. The occurrence of oxidative stress in light of rapid kinetic behavior upon administration was reported in established research. Similar observations were noted in monosodium glutamate (MSG), a compound with comparable pharmacokinetic behavior to MA. Repeated i.p. injections and oral intake of MSG induced oxidative stress, as evidenced by elevated levels of malondialdehyde (MDA) in rat thymus and kidneys [46–48]. However, the effect between single-dose exposure and oxidative burden formation is less explored. Therefore, to our knowledge, our study is to first to examine the time-course effect of single-dose oral exposure to MA on the kinetics of oxidant burden formation and clearance *in vivo*; since the excretion of these biomarkers in urine represents the average rate of oxidative and nitrative damage in the body, both of which are important factors in predicting disease development [49, 50]. Furthermore, our results demonstrated that regardless of sex, dose-dependent increases in the excreted levels of the 8-OHdG and 8-NO_2_Gua within 12 h of exposure. Current findings suggest that even after MA has been absorbed and excreted, the elevated levels of the abovementioned biomarkers persisted for days after dosing. As a result, we postulate that MA consumption may have weakened cellular antioxidant defenses, which is reflected in the increased formation of detectable 8-OHdG, 8-No_2_Gua and 8-isoPGF_2α_ in urine. Therefore, our results counter previous observations that MA induces neither oxidative stress nor inflammatory response and thus promote the concept that oxidative stress is, at least in part, responsible for MA-induced cytotoxicity.

8-isoPGF_2α_ is a biomarker of peroxidative attack of polyunsaturated fatty acids and membrane lipids [51, 52]. The statistically meaningful increase in 8-isoPGF_2α_ levels signals not only lipid peroxidation, but can also be a powerful predictor of kidney and lung diseases, since high urinary levels of 8-isoPGF_2α_ may also reflect cellular damage to the lungs [30]. Considering that the general population is likely to be exposed to MA via inhalation of automobile exhaust and aerosols [2, 3], future studies could examine whether chronic inhalation exposure to MA would also induce similar phenomenon observed in our study.

Existing research explored whether sex- and age-related differences contribute to variations in oxidative biomarkers [53, 54]. Some studies demonstrated that plasma concentrations of certain biomarkers, such as F2-isoprostanes, are higher in men than women, while others found that post-menopausal women have higher levels of urinary 8-isoPGF_2α_ compared to those who are premenopausal [55–57]. The aforementioned differences indicate that certain oxidant attacks, such as lipid peroxidation, may be more pronounced in males than females due to different expressions of antioxidant enzymes [58]. Although Brunelli and team reported that variations in oxidative stress biomarkers observed in rats are not attributed to sex-related differences [59], the preliminary results of our study revealed that, in male rats, urinary concentrations of 8-OHdG, 8-NO_2_Gua, 8-IsoPGF_2α_, are more likely to reach statistical significance compared to that detected in female rats.; previous research reported similar observations in urinary 8-OHdG levels in humans [60, 61]. Thus, our initial findings suggest that female rats may be less susceptible to oxidative stress incurred by MA, even though higher urinary creatinine levels of 8-OHdG and 8-IsoPGF_2α_ were detected in female rats. This observed phenomenon is in keeping with previously reported literature, which revealed that 8-OHdG levels are higher in females than males [62, 63]. Additional and more complex animal studies, along with different animal models and varied dosing regimen, analyzing biomarkers in plasma, kidneys and liver can lend credence to our findings.

The data presented in this study demonstrated that urinary concentrations of the four analytes from the treatment groups remained consistently higher than those from unexposed rats. Such results indicated that single-dose exposure lead to accumulation and persistence of these biomarkers, implying inadequate detoxification of MA at the cellular level and thus may signal the inception of cytotoxicity. Additional studies with a larger animal sample size could look into whether the abovementioned biomarkers can be detected in the liver or kidneys; future studies are needed to reveal conspicuous cellular structure alterations from both dose groups, since our initial histopathological observations fell short to reveal physio-morphological changes.

## Conclusion

The present study is the first, to our knowledge, to confirm that single-dose oral exposure to MA elevates urinary levels of 8-NO_2_Gua, 8-OHdG, and 8-isoPGF_2α_, which are representative biomarkers of oxidative and peroxidative damage, as well as inflammation. Our analysis also demonstrated that the elevated levels of the aforementioned biomarkers remain for days after one-time exposure. These findings promote the concept MA-induced hepato- and nephrotoxicity arise, at least in part, from oxidant and nitrative burdens. Considering the increase in urinary levels of HNE-MA was not statistically significant, and the histology readings were inconclusive, additional repeat-dose exposures to MA can help confirm such findings.

## Supporting information

S1 Fig Daily body weight change of maleic acid-treated Spague-Dawley male rats in all three dose groups. Dose groups receiving 0 mg/kg (●), 6 mg/kg (▼),and 60 mg/kg (■) are denoted accordingly.
(PDF)

S2 Fig Daily body weight change of maleic acid-treated Spague-Dawley female rats in all three dose groups. Dose groups receiving 0 mg/kg (●), 6 mg/kg (▼),and 60 mg/kg (■) are denoted accordingly.
(PDF)

S1 File S1_Figure_raw_data. Raw data points documenting average daily body weight changes of maleic acid-treated Spague-Dawley male rats in all three dose groups.
(JNB)

S2 File S2_Figure_raw_data. Raw data points documenting average daily body weight changes of maleic acid-treated Spague-Dawley female rats in all three dose groups.
(JNB)

S3 File Figure2a_2d_raw_data. Raw Data points analyzed via LC/MS/MS used to construct Fig 2A to 2D.
(ZIP)

S4 File Figure3a_3d_raw_data. Raw Data points analyzed via LC/MS/MS used to construct Fig 3A to 3D.
(ZIP)

## Abbreviations

8-OHdG: 8-hydroxy-2’-deoxyguanosine
8-IsoPGF_2α_: 8-iso-prostaglandin F_2α_
HNE-MA: N-acetyl-S-(tetrahydro-5-hydroxy-2-pentyl-3-furanyl)-L-cysteine
8-NO_2_Gua: 8-nitroguanine
LC-MS/MS: liquid chromatography tandem mass spectrometry
ROS: reactive oxygen species
RNS: reactive nitrogen species
ECD: electrochemical detection
ESI: electrospray ionization
HPLC: high-performance liquid chromatography
ID: isotope-dilution
MRM: multiple reaction monitoring
UV: ultraviolet
DDW: Distilled Deionized water
