# Correction: Elevation in and persistence of multiple urinary biomarkers indicative of oxidative DNA stress and inflammation: Toxicological implications of maleic acid consumption using a rat model

**DOI:** 10.1371/journal.pone.0214188

**Published:** 2019-03-20

**Authors:** Charlene Wu, Hsin-Chang Chen, Shu-Ting Chen, Su-Yin Chiang, Kuen-Yuh Wu

The authors wish to clarify the relationship between this *PLOS ONE* article (a single-dose study) [1] and a closely related repeated-dose toxicity study published in July 2017 in the *Journal of Applied Toxicology* [2]. Findings discussed in this *PLOS ONE* article [1] focus on oxidative stress induction and persistence after single-dose oral (gavage) exposure to maleic acid (MA), elucidating the kinetics of oxidant burden formation and clearance. In the *Journal of Applied Toxicology* study [2], the authors investigate the effects of repeated exposure to MA in rats, using ^1^H nuclear magnetic resonance analysis to focus on changes in the metabolic profile.

The single-dose study examines the formation and clearance of oxidative and nitrative stress-related biomarkers, while the repeated-dose study further investigates the accumulation and persistence of those biomarkers. Both studies report increased urinary levels of 8-OHdG, 8-NO_2_Gua, and 8-isoPGF_2α_ after MA exposure; the repeated-dose study also reports changes in alanine, acetoacetate, hippurate, succinate and acetate, indicative of hepatotoxicity

Furthermore, the authors would like to correct Fig 1, which was published in the supplementary material of the repeated-dose study in [2]. The revised Fig 1 now includes original data from the chromatogram that best demonstrate simultaneous quantification of the four biomarkers and their respective isotopes, identified as per a previously published protocol [3], representing a rat urinary sample collected at 0.5 days after a single dose of MA (60 mg/kg). Please see the correct Fig 1 and updated Fig 1 caption here.

**Fig 1:**
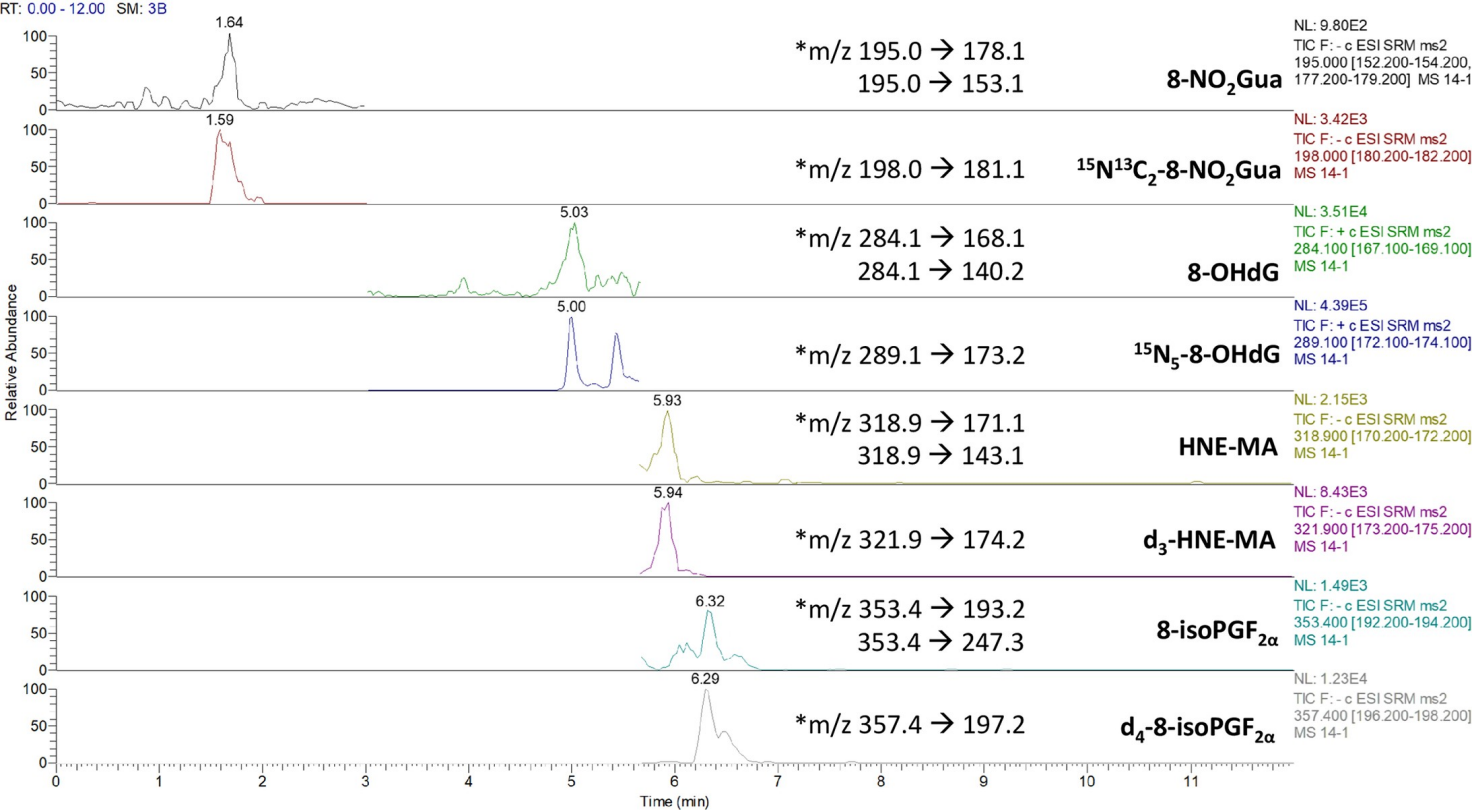
A chromatogram derived from analysis of a rat urinary sample collected at 0.5 days after a single dose of maleic acid (60 mg/kg) and analyzed using SPE-LC-MS/MS. Quantitation channels are marked with an asterisk (*).

To further clarify, this single-dose study was designed to evaluate the toxicokinetic profiles of toxicants and their biomarkers over time, rather than to investigate physio-morphological changes from repeated dosing. In fact, the authors found no significant histological changes in liver and kidney tissue at seven days in rats administered a single dose of MA as compared to untreated controls. The authors mention “histopathological observations” in the last sentence of the Discussion in [1], referring to results of analyses that were not included in the manuscript. The latter part of the last sentence should therefore be omitted. The correct sentence is: Additional studies with a larger animal sample size could look into whether the abovementioned biomarkers can be detected in the liver or kidneys; future studies are needed to reveal conspicuous cellular structure alterations from both dose groups.

Following this line of reasoning, the authors need to amend the wording of the Conclusion to avoid any mention of histology readings. In light of the repeated-dose study being published prior to the single-dose study, the Conclusion should read:

This time-course study confirms that oral single-dose MA exposure elevates urinary levels of 8-NO_2_Gua, 8-OHdG, and 8-IsoPGF_2α_, biomarkers that represent oxidative and peroxidative stress, as well as inflammation; these elevations tended to persist for some days. These results prompted us to conduct a repeated-dose study to investigate accumulation of these biomarkers and early pathological findings, to clarify the underlying mechanisms associated with oxidative and nitrative stress.

The authors confirm that these modifications do not alter the conclusions.
