# Supplementary figures and images for: Elevation in and persistence of multiple urinary biomarkers indicative of oxidative DNA stress and inflammation: Toxicological implications of maleic acid consumption using a rat model
Source: PLoS One. 2017 Oct 26;12(10):e0183675. doi: 10.1371/journal.pone.0183675 (PMC5658196; doi:10.1371/journal.pone.0183675)

S1 Figure

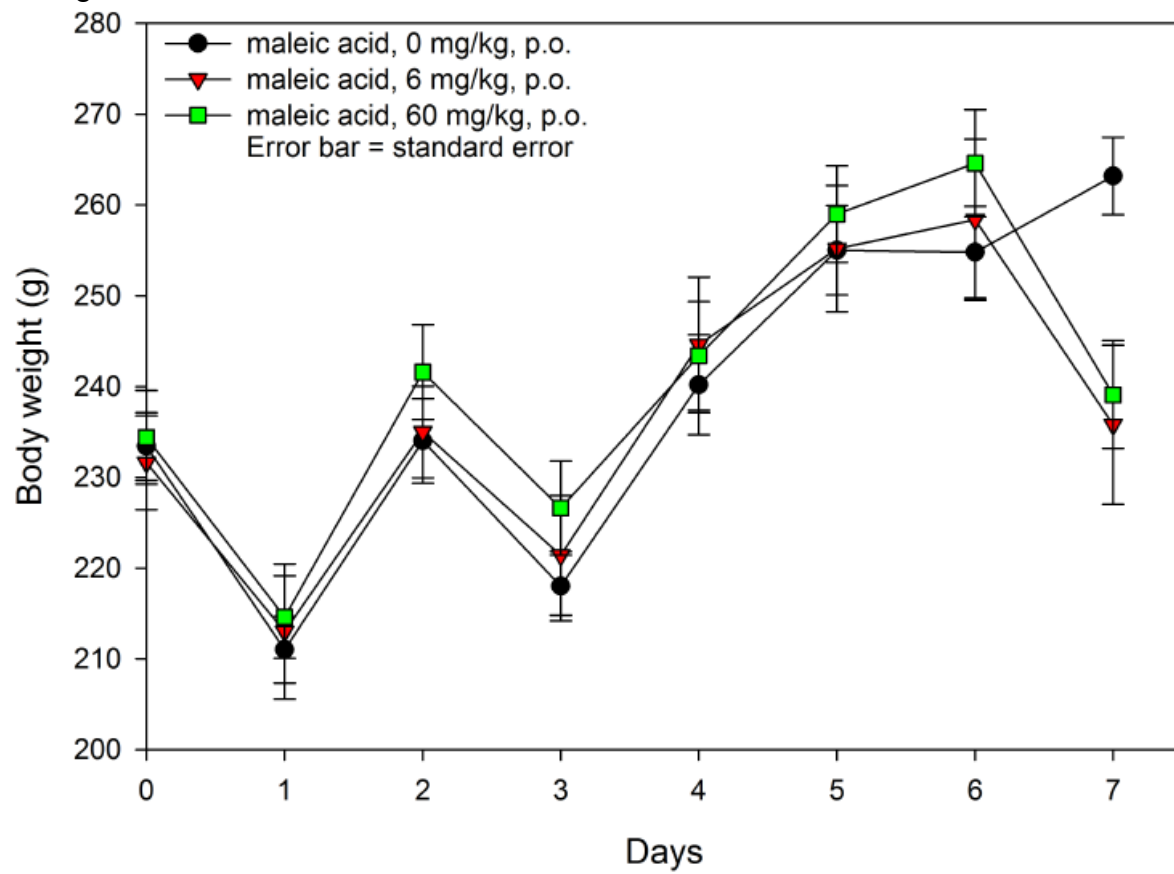

Supplement: S1 Fig — Dose groups receiving 0 mg/kg (●), 6 mg/kg (▼),and 60 mg/kg (■) are denoted accordingly. (PDF) [file pone.0183675.s001.pdf]

S2 Figure

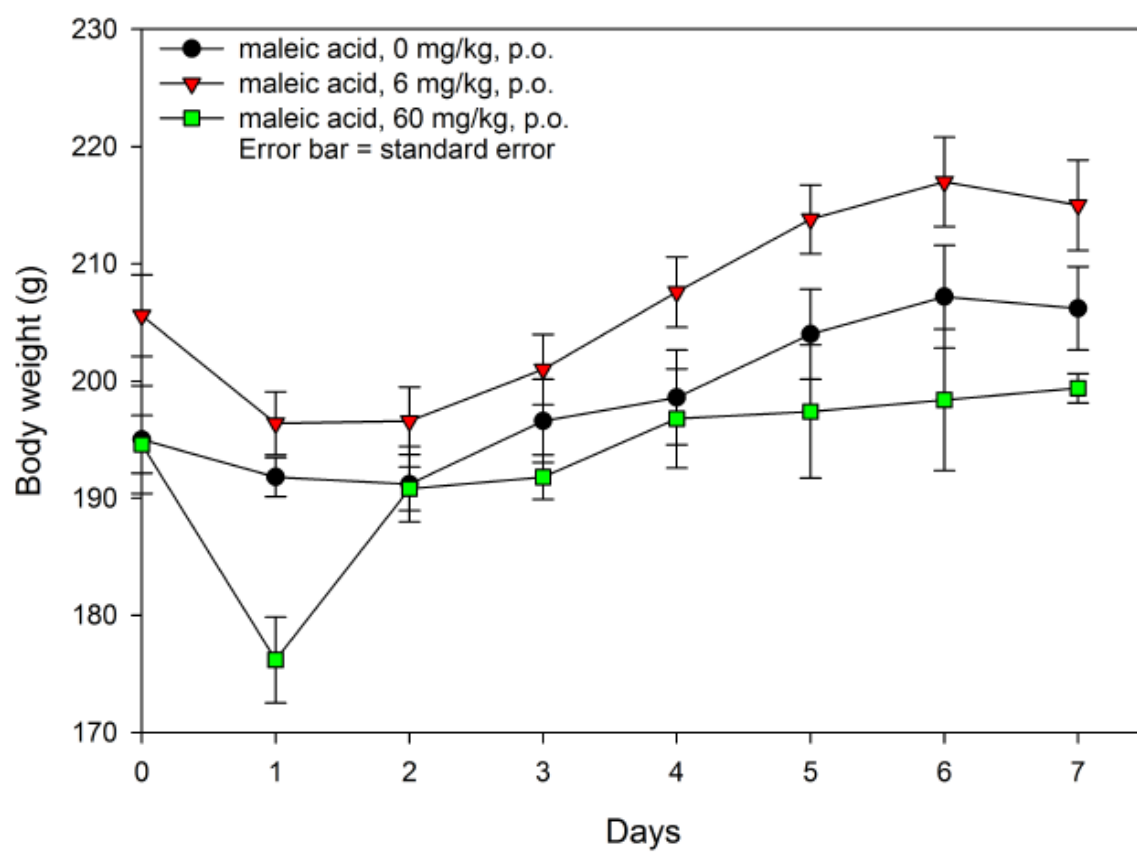

Supplement: S2 Fig — Dose groups receiving 0 mg/kg (●), 6 mg/kg (▼),and 60 mg/kg (■) are denoted accordingly. (PDF) [file pone.0183675.s002.pdf]
